# A brief 5-item version of the Neck Disability Index shows good psychometric properties

**DOI:** 10.1186/1477-7525-11-108

**Published:** 2013-07-01

**Authors:** David M Walton, Joy C MacDermid

**Affiliations:** 1 School of Physical Therapy, Western University, 1201 Western Rd, London, Ontario, Canada; 2 School of Rehabilitation Science, McMaster University, Hamilton, Ontario, Canada; 3 Clinical Research Lab, Hand and Upper Limb Centre, St. Joseph’s Hospital, London, Ontario, Canada

## Abstract

**Background:**

The purpose of this secondary analysis of clinical databases of people with neck pain was to use a mixed unique conceptual and statistical approach to develop a brief version of the Neck Disability Index (NDI).

**Methods:**

An a priori framework of neck-related function based on the International Classification of Functioning, Disability and Health was used to identify items from the original 10-item NDI that do not conceptually fit. Remaining items were subject to Rasch analysis to identify items that did not statistically fit with axioms of quantitative measurement. Finally, approaches drawn from classical test theory were used to compare stability, responsiveness and concurrent validity of the original NDI, the new brief NDI and the linearly-transformed brief NDI.

**Results:**

Conceptual analysis identified 3 items that did not fit with the construct of self-reported ability to perform activity: pain intensity, headache, and sleeping. These items were removed, and responses to the remaining 7 items drawn from an assembled database of 316 physiotherapy patients with neck pain were subject to Rasch analysis. Two items were removed due to either considerable differential item functioning (reading) or statistical redundancy (lifting). The remaining items were considered the NDI-5. Test-retest reliability, responsiveness, sensitivity to change, and concurrent validity were all comparable across the original NDI, NDI-5 and linearly-transformed NDI-5. Sensitivity to change over a 1-month period of physiotherapy was the notable exception, where the linearly-transformed NDI-5 showed superiority over the other two forms.

**Conclusions:**

A shortened version of the NDI, the NDI-5, has been constructed that is conceptually and statistically sound. Implications for research and clinical practice are discussed. Comparison with the NDI-8 is provided that suggests overall similar function across the forms, although the latter may be more sensitive to change.

## Background

Patient-reported outcomes (PROs) are increasingly becoming a routine part of clinical practice. Such tools can be useful for prognosis, treatment planning, evaluating response to treatment, and determining suitability for discharge. In the area of neck pain specifically, a recent international survey of 381 clinicians [1] found that 75% of respondents used a pain intensity rating scale routinely, and 49% used the Neck Disability Index (NDI, [2]) sometimes or routinely. A scoping review of the scientific literature found the NDI to be the most commonly used neck-specific measure of disability [3] and a separate review of prognostic literature following whiplash injury also found that, amongst disability-centric rating scales, the NDI was the most commonly used tool to identify ‘recovery’ [4].

Despite widespread use, the NDI has not been consistently endorsed as a strong psychometric tool, especially when subject to rigourous evaluation. [5] used Rasch analysis to determine, among other properties, that the NDI suffers from lack of unidimensionality, which poses conceptual problems when the scale is intended to provide a summative score across all items. This violates a fundamental axiom of quantitative measurement [6], that being that any scale which is meant to be summed and subject to mathematical procedures should measure only a single dimension. Further, if the scale is to be subject to statistical analyses that assume interval-level (linear) measurement, such as comparison of group means or correlational analyses, it should conform to linear measurement models, such as that offered by Rasch analysis [7]. From a clinical perspective, pain and function are generally considered two different constructs, and are amenable to different types of intervention. The inclusion of both constructs in a single scale risks masking important effects of treatment on one construct if the other remains stable or increases, as in the case of increased function at the expense of increased pain or vice versa. The result of the van der Velde et al. [5] analysis was endorsement of an 8-item version of the NDI that conformed adequately to the Rasch model.

Beyond statistical concerns, the NDI also represents a time burden that may be a barrier to wider adoption in clinical environments. One of the most commonly reported barriers to use of PROs or implementation of other best practice guidelines is a perception of the burden of time required to do so properly [8,9]. The original NDI comprises 10 domains, each with 6 statements that are to be read by the respondent, who then chooses the one statement that most closely represents his/her current functional status within each domain. This means that, in order to complete the NDI correctly, the participant must read 60 separate statements, which is considerably greater than many other common region-specific disability scales such as the Roland-Morris low back disability questionnaire (24 statements, [10]), the Lower Extremity Functional Scale (20 statements, [11]), and the Disability of the Arm, Shoulder and Hand scale (30 statements in base scale, [12]). A review of the properties of the NDI found evidence to suggest that time to completion of the scale ranges from 4 to 9 minutes [13] verging on too long for routine clinical use. Even the NDI-8 of van der Velde and colleagues [5], while psychometrically more sound than the original, requires 48 statements be read.

The purpose of this study was to determine whether an even shorter, ‘brief’ version of the NDI could be created that possessed psychometric properties comparable to the original while focusing on a single construct (function) and reduced response burden. This is in contrast to the stated purpose of van der Velde and colleagues [5], who attempted to improve the psychometrics of the NDI using the least amount of modification necessary. The major conceptual difference is that our goal was to create the shortest possible scale that remained conceptually and statistically sound. Both qualitative (descriptive theoretical analysis) and quantitative (Rasch, classical test theory) approaches were used to arrive at a brief NDI.

## Methods

The process of creating a brief NDI started with detailed conceptual analysis of each domain on the original NDI. The current International Classification of Functioning, Disability and Health [14] served as a conceptual framework. The ICF describes health as influenced by normal biological tissue structure and function, capacity to perform activities, and participation in valued life roles, influenced by personal and environmental factors. Owing to a desire for a self-reported function-based neck disability questionnaire, those items that fit within the ‘activity’ domain of the ICF were targeted for retention.

For quantitative analysis, patient data from 4 independent cohorts of community-based outpatient physiotherapy patients with mechanical (specific or non-specific) neck pain were compiled to provide a database of 316 patients. Subjects in all cohorts were eligible if they presented to physiotherapy for reasons pertaining to neck pain that could not be attributed to fracture, dislocation, cancer or other systemic disease (e.g. rheumatism), were between 18 and 70 years old, and could speak and understand conversational English. Patient characteristics included: sex, age, duration and cause of symptoms, and medicolegal status (litigation and/or compensation engagement). Work status was collected in a subset of 212 respondents and was categorized as normal duties, neck-related sick leave, or other type of leave. Self-reported indicators of pain and psychological components included: the Pain Catastrophizing Scale [15] the Tampa Scale for Kinesiophobia 11-item version [16] and the Numeric Pain Rating Scale [17]. The PCS, TSK and NRS have demonstrated adequate test-retest reliability (ICCs generally exceeding 0.76) and validity in people with neck pain [18-20]. When items were missing, cases were dropped for that analysis and the sample size was modified accordingly.

To assess longitudinal validity, NDI and NRS scores were obtained on multiple occasions from a non-random sub-sample of 50 subjects undergoing physiotherapy treatment for their neck problems. These subjects completed the NDI, NRS and a Global Perceived Rating of Change scale (GPRC, ranging from -7 = A very great deal worse, through 0 = no different, to +7 = A very great deal better) on a weekly basis for 1 month. The treatment was individualized to the patient and included any or all of advice, education, manual joint mobilization, soft tissue stretching, therapeutic modalities and/or therapeutic exercise.

All methods were approved by the Institutional Review Board at Western University prior to initiating data collection in each study cohort.

### Analysis

#### ***Item reduction***

The process of shortening the NDI was accomplished through a combined qualitative and quantitative approach, guided by existing theory and the ICF conceptual framework of health and disability. The lead author, an experienced clinimetrician with over 10 years clinical experience in treating people with neck pain, conducted the first pass identification of conceptually misfitting items i.e. those that did not fit with the definition of disability (activity/participation) as defined by ICF. The second author, also an experienced clinimetrician and clinician with extensive knowledge of the ICF, independently classified items as fitting within or outside of the disability domain. Upon consensus, only disability-related items were retained for further analysis.

The remaining items were subject to Rasch analysis to establish the degree to which the scale conforms to axioms of quantitative measurement [7]. The steps taken in Rasch analysis can be extensive and have been detailed elsewhere [20,21]). In brief, Rasch analysis assigns each item and each respondent (termed ‘person’) a location along a unitless logit-transformed continuum of the construct under measure, in this case disability. Rasch analysis posits that the relative location of an item along the continuum is estimable by virtue of knowing only the relative location of the person answering it. Conversely, the relative location of any person should be estimable by virtue of knowing only their response to an item. The error between the observed and estimated locations is termed a ‘residual’, and the magnitude of all residuals can be tested using a bonferroni-corrected χ^2^ test to determine whether the observed locations deviate from the estimated locations to a degree greater than chance, which would indicate misfit of the scale to the Rasch model. Rasch analysis provides the important advantage of allowing deeper exploration of misfitting items to identify the cause of misfit. Causes include multidimensionality, location dependence, disordered response thresholds, or differential item functioning, as outlined below [21,22].

Multidimensionality and location dependence are often evaluated together. A correlation matrix of residuals for each item is generated, and patterns of strong correlations (r > 0.20 points above the mean correlation) suggest the residuals (error) are not random but rather shared (systematic) across items. Strong item residual correlations indicate location dependence or multidimensionality (subgroups of items) within the scale. The residuals can also be factor analyzed using principal components analysis. Two subsets of items can be generated; those that load positively and those that load negatively on the first factor. A logit-transformed score is calculated for each person using both subsets of items, and differences between scores attained from each subject in the database are evaluated using a t-test. If less than 5% (<16 respondents in this case) of those t-tests are significantly different at the p<0.05 level, and no obvious pattern of residual correlation is identified, then the assumptions of unidimensionality and location independence are satisfied.

Disordered response thresholds are identified by plotting the likelihood of choosing each possible response to an item along the continuum of disability. The ‘threshold’ is that point along the continuum where two adjacent responses are equally likely (50% chance each). If the threshold for two items, say a 2 or a 3 on the NDI, is located higher than the threshold of two adjacent items, say a 3 or a 4 in this example, then the thresholds are considered disordered. Disordered response thresholds may be an indication of ambiguous or unclear wording, or gradations that are too fine leading to an inability of respondents to reliably choose a response. Collapsing scores across disordered items is a sound way of addressing this problem.

Differential item functioning (DIF) is explored through an ANOVA technique. The sample is separated into *class intervals*, or subgroups of respondents at similar relative locations, which serve as the within-groups factor. The between-groups factor is level of the variable being explored, which in this case were: sex (male/female), cause (traumatic/nontraumatic), duration (less than 6 months, 6 months or greater) and medicolegal status (engaged in litigation or compensation/ not engaged). DIF can be uniform, where the between-groups factor is significant across all levels of disability, or non-uniform, where the factor only influences responses at a certain level of disability. The best approach to dealing with DIF is determined on an item-by-item basis. Once all assumptions of quantitative measurement have been satisfied, and the scale items represent a reasonable ‘ruler’ of the construct to be measured (disability), a transformation matrix can be constructed which allows conversion of ordinal-level responses to interval-level responses using the logit-transformed scores.

### Responsiveness and stability

The items remaining after the conceptual and Rasch analyses were considered a prototype ‘brief’ NDI and the properties of both the raw ordinal-level scores and transformed interval-level scores were subject to comparison with the properties of the original 10-item scale. Test-retest reliability over a 1-week period for each of the full NDI, brief NDI and linear (logit)-transformed brief NDI was evaluated in the repeated measures subgroup using the Intra-class correlation coefficient Type 2,1 (ICC_2,1_, [23]). Data from only those subjects who reported no clinically meaningful change in their neck problems over a one week period (GPRC of -1, 0, or +1) were used for this analysis.

Ability to detect clinically meaningful change was evaluated by separating the longitudinal sample into two groups: those that had changed a clinically important amount and those that had not over 1 week of treatment. A receiver operating characteristic curve (ROC, sensitivity plotted against 1-specificity) was constructed for each version of the scale (full, brief ordinal, and brief linear) and the area under the curve (AUC) was used as the indicator of responsiveness. Meaningful change was defined as an absolute GPRC change score of at least 2 points (a little change) up to 7 points (a very great deal of change) over the first week of treatment. An area under the curve (AUC) of 0.50 indicates that the scale was no better than chance at discriminating between those who had and those who had not changed a subjectively meaningful amount, while an AUC of 1.0 indicated perfect discriminative ability.

The effect size of the 3 NDI versions (full, brief ordinal, brief linear) for evaluating change with the physiotherapy treatment group (n = 50) over 4 weeks was calculated by dividing the mean difference between baseline and 4-week follow up by the standard deviation of the baseline score. Effect size (ES) was interpreted as small = 0.2, medium = 0.4, and large = 0.8 [24]. For ICC_2,1_, AUC, SEM, Pearson’s r, and ES, 95% confidence limits were calculated to determine whether the point estimates were significantly different across the 3 versions of the NDI.

### Concurrent validity

Concurrent validity was evaluated through Pearson’s r between the 3 versions of the NDI and the concurrently-measured NPRS, TSK-11 and PCS

## Results

**Figure 1:**
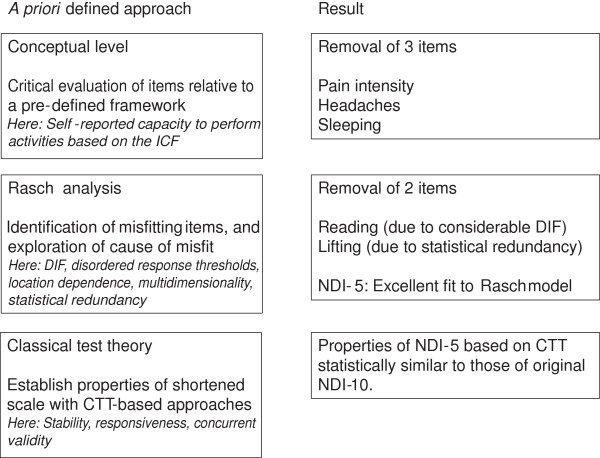
Frame work for developing the shortened scale.

Figure 1 presents the process of scale shortening in a graphical format.

### Conceptual analysis

The World Health Organization recognizes the complexity of disability, defining it as an umbrella term that integrates a health condition within personal and environmental factors. (http://www.who.int/topics/disabilities/en/). The ICF model draws a distinction between *capacity* to perform (‘can do’) and *actual* performance (‘do do’). Through scrutiny of the items on the NDI, it is focused on the self-reported beliefs in one’s capacity to perform a number of functional activities (‘can’ do), some specific (lifting, reading, driving), some generic (work, recreational activities). The two notable exceptions are items 1 (pain intensity) and 5 (headaches), both symptom-based items that do not inherently measure function. The headache item is a notable challenge, in that the 6 response options each include both an intensity (no, slight, moderate, severe) and frequency (no, infrequently, frequently, all the time) qualifier. Combining intensity and frequency will result in ambiguity for some people, for example, those with severe headaches that come infrequently. Owing to our purpose of developing a unidimensional, brief, valid scale of function that would be sensitive to physical (conservative) rehabilitation, the two symptom-based items were removed.

Item 9 (sleeping) is the only item on the scale that is performance-based (actual hours of disturbed sleep) rather than capacity-based. Taking the perspective of improving assay sensitivity it was judged that while sleep is important for people with neck pain, it was not necessarily linked to function-based rehabilitation programs and might be more aligned with symptoms. Considering our desire to create a scale that reflects functional levels and is responsive to changes in function, this item was removed. A sensitivity analysis (not shown) indicated that removal of this item did not influence the results of the Rasch analysis below in any meaningful way.

### Rasch analysis

For purposes of comparison with van der velde and colleagues, [5] we first performed the Rasch analysis using the full set of 10 items on the database of 316 subjects after removing 7 extreme (floor) scores. Overall the scale showed poor fit to the model (χ^2^ = 89.1, p<0.001). Removing the 3 items (pain, headaches, sleeping) led to good fit (χ^2^ = 37.2 p = 0.24) with mean item location of 0.00 (SD 0.81) and mean person location of -0.18 (SD 1.30), suggesting good targeting of the sample to the scale. Tests for unidimensionality and location dependence suggested that two items (driving and lifting) were co-dependent by virtue of a strong residual correlation, and were statistically redundant loading at -0.51 (lifting) and -0.59 (driving) logits, respectively. Item 8 (driving) showed disordered response thresholds, where the threshold between 3 and 4 was located higher than that for 4 and 5. Looking at the response options for that item, option 3 (I can’t drive my car as long as I want because of moderate pain in my neck) and 4 (I can hardly drive at all because of severe pain in my neck) are both tapping a driving restriction somewhere between none (option 2) and complete (option 5). Conceptually, collapsing these two response options would still provide an indication of restricted driving but lose the precision of neck pain severity, which is best left to dedicated pain severity scales. For this reason, options 3 and 4 of item 8 were collapsed, and that item was rescored 0-1-2-3-3-4, while the original scoring structure of 0-1-2-3-4-5 was retained for all others. Item 4 (reading) showed evidence of uniform DIF for each of sex, duration of symptoms and medicolegal status. One method to address DIF is to split the item, essentially creating different scales for males & females, acute & chronic pain, or those involved & not involved in medicolegal processes. Another option is to reword the item to make it more relevant to all subclasses, or a third option is to remove the item outright. Owing to our goal of a simplified tool, and because the conceptually similar item 6 (concentration) was retained, we opted to remove item 4 at this stage.

The problem of location dependence and redundancy between driving and lifting was solved as follows: Both are potentially important, although driving has been rated as more important in focus groups according to both Hoving and colleagues [25] and En and colleagues [26]. A critical evaluation of each item revealed both suffered from conceptual ambiguities, although collapsing the response options of the driving item as above addressed concerns for that item. The fit to the Rasch model with both items retained (χ^2^ = 37.2 p = 0.24), only lifting (χ^2^ = 21.5 p = 0.37) or only driving (χ^2^ = 23.6 p = 0.26) suggested that fit was not adversely affected when only 1 of the 2 were retained. A sensitivity analysis of stability, responsiveness and convergent validity (not shown) indicated nearly identical overall scale function when only driving, only lifting, or both were retained. These results suggest that only 1 of the 2 needed to be retained, and in accordance with the findings of the previous focus group studies, the driving item was retained but rescored as above, while the lifting item was removed.

**Figure 2:**
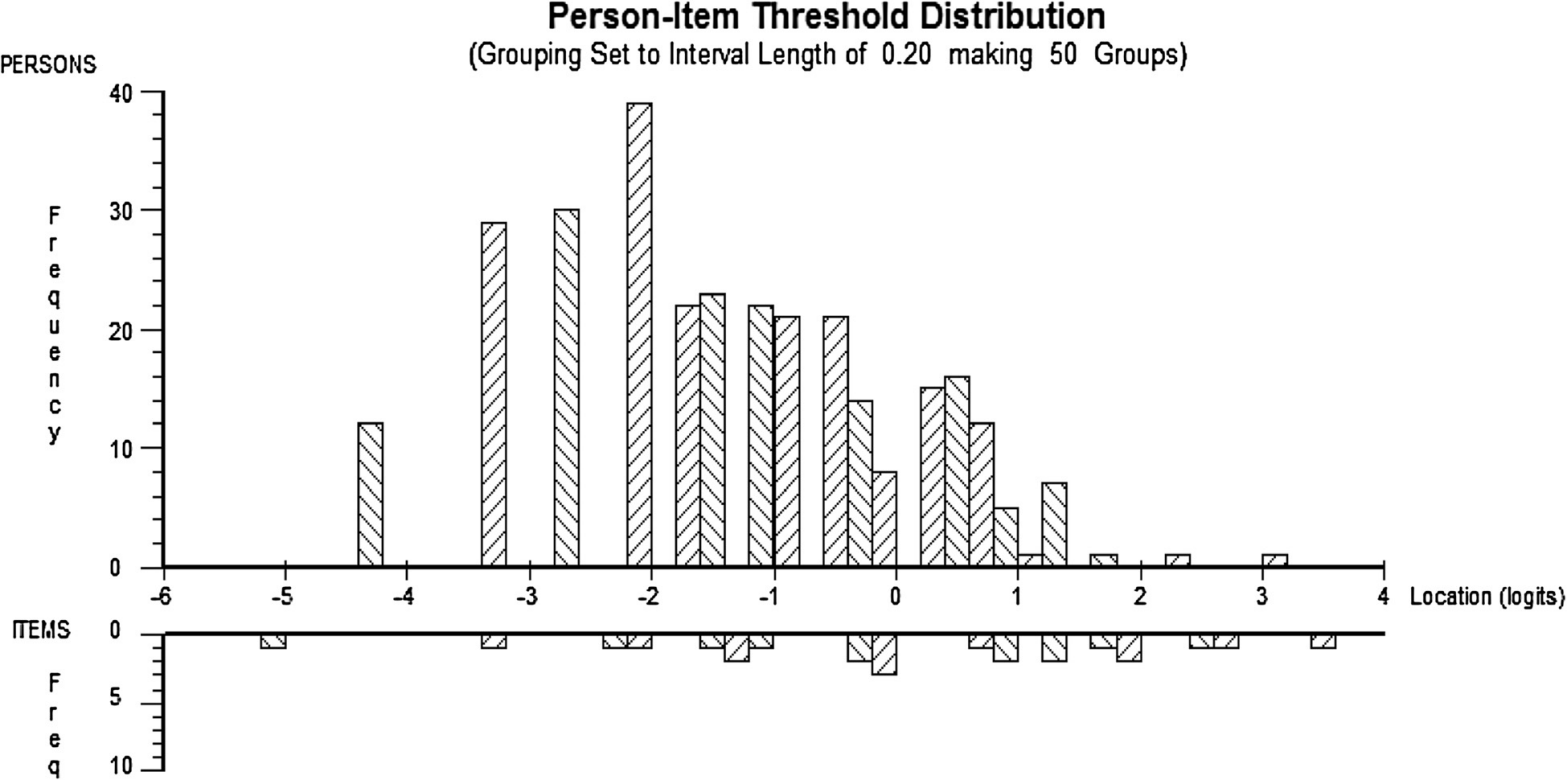
Person in item threshold developing of NDI-5.

This process lead to a 5-item version of the NDI with satisfactory indicators of location dependence and unidimensionality (4.6% significant t-tests, mean residual correlation of 0.19, range 0.08 to 0.33). The brief-NDI, from here forward referred to as the NDI-5, included the following items (in order of difficulty): Person care, Concentration, Work, Driving (rescored), and Recreation. The scale range was therefore 0 to 24. This version of the NDI showed excellent fit to the Rasch model (χ^2^ = 23.6, p = 0.26), a mean item fit residual of 0.0 (SD 0.85), and a person separation index (PSI) of 0.79. Removal of additional items led to a PSI of <0.70, inadequate for individual or group use. Figure 2 shows the histogram of person to item threshold locations, suggesting the 5-item version of the scale adequately covers the distribution of respondents in our sample, although it may be deficient for capturing change at very low levels of disability. A transformation matrix for converting raw ordinal to interval-level scores out of 24 and out of the more recognizable 50 is included in the Additional file 1.

### Stability

Table 1 shows the test-retest reliability estimates of the original 10-item version, the brief NDI-5 and the linearly-transformed brief NDI-5 (from here on referred to as NDI-5 linear) along with the standard error of measurement (SEM) and minimum detectable change at the 90% confidence level (MDC_90_). Reliability estimates ranged from ICC_2,1_ = 0.89 (NDI-5 linear) to 0.92 (original), with 95% confidence limits suggesting no significant difference across all 3. One-week MDC_90_ for all 3 versions, expressed as a percentage of overall scale score, was 9% (NDI original), 11% (NDI-5) and 10% (NDI-5 linear).

**Table 1:** **Relative (ICC) and absolute (MDC**_**90**_) reliability estimates over 1 week*

	**ICC (95% CI)**	**SEM (95% CI)**	**MDC**_**90**_
NDI10	0.92 (0.85, 0.96)	1.95 (1.76, 2.67)	4.5
NDI5	0.91 (0.83, 0.96)	1.15 (0.95, 1.45)	2.7
NDI5 linear	0.89 (0.80, 0.95)	1.05 (0.98, 1.48)	2.4

*: Only those subjects who indicated a stable condition over this period, as indicated by a GPRC of -1, 0, or +1, are included in this analysis (n=36).

### Responsiveness

Table 2 provides the AUC for discriminating between those who had and had not changed over a 1 week period. The AUC ranged from 0.71 (NDI-5) to 0.76 (original). All estimates were significantly greater than chance, and were not statistically different from each other. Table 2 also provides the calculated effect sizes for the 3 scales following 1 month of personalized physiotherapy intervention. The NDI-5 linear showed the highest responsiveness (0.87) while the NDI-5 ordinal showed the lowest (0.56). Broad confidence intervals indicate that the estimates of effect size were not statistically different from one another but also limit confidence in the accuracy of the point estimate.

**Table 2:** Responsiveness estimates of the 3 versions of the NDI

	**AUC (95% CI)**	**ES (95% CI)**
NDI	0.76 (0.63, 0.89)	0.71 (0.40, 1.02)
NDI-5 ordinal	0.71 (0.57, 0.85)	0.57 (0.27, 0.86)
NDI-5 linear	0.72 (0.59, 0.86)	0.85 (0.53, 1.17)

*AUC* area under the receiver operating characteristic curve for identifying a clinically meaningful changed defined as a GPRC of -2 or lower, or +2 or higher. *ES* effect size over 1 month of physiotherapy treatment [(mean post – mean pre) / SD pre].

### Cross-sectional validity

Table 3 provides the correlation coefficients between the 3 versions of the NDI and each of NPRS, TSK and PCS. All associations were of expected direction and magnitude and all were statistically significant (p<0.05). Estimates across all 3 versions were also very similar with no version showing clear superiority over the other two.

**Table 3:** Cross-sectional correlations between the 3 versions of the NDI and key measures. All associations are significant at the p<0.01 level

		**r (95% CI)**	
	NDI	NDI-5	NDI-5 linear
NPRS (n = 309)	0.71 (0.49, 0.85)	0.67 (0.42, 0.82)	0.69 (0.45, 0.84)
TSK (n = 261)	0.53 (0.23, 0.74)	0.54 (0.24, 0.75)	0.56 (0.27, 0.76)
PCS (n = 153)	0.64 (0.38, 0.81)	0.64 (0.38, 0.81)	0.59 (0.31, 0.78)

*NPRS* Numeric Pain Rating Scale, *TSK* Tampa Scale for Kinesiophobia-11 items version, *PCS* Pain Catastrophizing Scale.

### Comparison with NDI-8

For the sake of comparison, we calculated the NDI-8 score for each subject based on the findings of van der velde and colleagues [5] and compared the key indicators between the original NDI, NDI-8 and our NDI-5. The magnitude of correlation between the NDI-8 and each of the NPRS, TSK and PCS (r = 0.72, 0.53, 0.65, respectively) were nearly identical to those of the NDI and NDI-5 (see Table 3). The AUC of the NDI-8, indicating its ability to discriminate between those who had and those who had not changed a meaningful amount, was 0.79 (95% CI 0.66, 0.91), comparable to the NDI (0.76, 95% CI 0.63, 0.89) and NDI-5 (0.71, 0.57, 0.85). Finally, the effect size calculated over the course of 1 month of physiotherapy treatment was 0.69 (95% CI 0.38, 0.99) for the NDI-8 ordinal and 1.13 (95% CI 0.78, 1.48) for the NDI-8 linear.

## Discussion

We have described a novel qualitative and quantitative approach to arriving at a conceptually and statistically sound brief version of the NDI, the NDI-5. The properties of the NDI-5 are statistically similar to the original 10-item version for all indicators explored, while the linear transformation may provide slightly greater sensitivity to change. The reduced number of items offers the important clinical advantage of reduced time burden while still offering measurement properties that are meaningful to clinicians.

The properties of the NDI-5 are comparable to those of the NDI-8 [5] although the latter may be more sensitive to change. The effect size of the NDI-8 linear appears to be greater than that of the NDI-5 ordinal and the original NDI (lower limit of 95% CI = 0.78 for the NDI-8 linear vs. point estimate of ES = 0.57 and 0.71 for the NDI-5 ordinal and for the original NDI, respectively). No other comparisons suggested a significant difference in this regard by virtue of overlapping confidence intervals.

Both compare favourably with the original NDI, and we hold the belief that by virtue of removing the symptom-based items, the NDI-5 is more conceptually sound. This is of course a matter of opinion; the inclusion of symptoms may contribute to the NDI – 8 being more responsive. The contention being put forth here is that pain and function, while related, are distinct constructs. Symptoms are clinically important and should be tracked separately, and symptoms and function are likely to respond differently to a given intervention. Where an intervention, such as strengthening, is intended to improve function, simultaneously capturing symptom-based items may confound the magnitude of effect. Similarly, an intervention meant to improve pain, such as opioid medication, may not have the same magnitude of effect on function. In some cases an intervention meant to improve function may in fact worsen pain, or vice versa. Such an effect would be masked by a multidimensional scale. Where both are desirable as patient-reported outcomes, a logical suggestion would be to use a scale such as the NDI-5 or other function-based scale to capture function, and a validated symptom-based scale (such as a dedicated Numeric Pain Rating scale) to capture symptom intensity, and to evaluate the two separately in order to maximize assay sensitivity.

Rasch analysis provides detailed evaluation of scale properties, down to the function of individual response options, while classical approaches (reliability, convergent validity) provide support for the properties of a scale that are easily interpretable by clinicians. The Rasch approach also offers the added benefit of a transformation matrix which allows ordinal-level scores to be converted to interval-level scores. This conversion is especially relevant to research applications, where statistical assumptions often require that data be interval level. The transformation has clinical implications as well; the transformation matrix suggests that the amount of linear change is not uniform across the entire breadth of the scale, and that ordinal-level changes at the poles may be more meaningful on a point-for-point basis than are similar changes in the mid-portions of the scale. This general relationship was also reported for the NDI-8 [5], having now been independently validated in our study.

The magnitude of association between the different versions of the NDI and other constructs such as pain, catastrophizing, and fear are generally in keeping with previous work in the field [27,28] and suggest that the construct(s) being captured across all versions are similar. The decision regarding which of the original NDI, NDI-8 or currently described NDI-5 to use must be based on the use scenario: both the NDI-8 and NDI-5 conform better to the Rasch model of interval level measurement than the original, and both are conceptually sound. By incorporating pain intensity into the functional scale, the NDI-8 may be slightly more responsive to change than the NDI-5, but also risks confounding functional change with symptomatic change. Both appear to be associated with the same constructs, and both are adequately reliable and responsive for routine clinical use.

Readers should note that the removal of items pertaining to pain, headaches, lifting, reading and sleep should not be taken to suggest that these domains are unimportant. However, for either conceptual or statistical reasons, they either do not fit with the construct of neck-related function, do not function well in their current form, or are statistically redundant and therefore do not add enough additional information to warrant retention. Each of these constructs have been deemed more or less important to the neck pain experience by previous authorship groups [25,26]. As mentioned above, pain and other symptoms might be important enough to warrant a scale dedicated to their measurement. Similarly, sleeping is adequately important that a sleep-specific scale, such as the Pittsburgh Sleep Quality Inventory, [29] may be a more valuable approach to its measurement. Lifting appears to be an important function for people with neck pain, but its inclusion on the NDI did not offer any improvement in measurement properties. It is possible that this is because the lifting item in its current form is taken almost verbatim from the low-back-specific Oswestry Disability Index, [30], and as such the focus is on lifting items from waist level or below. People with neck pain may well have problems with lifting or reaching, but these problems are likely to manifest when the activity is performed at or above shoulder level rather than below waist level. Inclusion of a refined version of this item may lead to better properties than what have been reported here.

This leads to an important consideration; we have addressed problems with item misfit primarily through item removal and re-testing of the scale in the same cohort. This was an intentionally liberal approach given our goal of creating the shortest scale possible. While we’ve determined that the NDI can be reduced as far as 5 items and still function adequately well, an alternative and arguably better approach would have been to re-word certain items or define new formatting for the scale and then re-test it in an independent sample. This appears to be a ripe direction for future research, and one that is sorely needed considering the influence on policy that research findings using the NDI may have (for example: [31]).

Shortening and homogenizing has both advantages and disadvantages. Whilst the scale may well be conceptually sound, it does mean that some items of importance to people with neck pain will be missing. This argument is not unique to the NDI-5, it also holds for the NDI-8, original NDI, and any scale with defined activities. While there is value in standardized scoring tools, especially for comparison across patients or research samples, patient-centred outcomes are also valuable. For this reason clinicians are encouraged to additionally consider the use of scales such as the Patient-Specific Functional Scale [32] or Canadian Occupational Performance Measure [33], both of which allow respondents to generate their own areas of concern and weight them accordingly.

We have addressed a number of limitations to this study already, most notably that item removal was liberal and that other authorship groups may have arrived at a different scale than what has been presented here. Readers will also note that the driving item has been modified slightly on the NDI-5 to read ‘driving / riding in a vehicle’, in recognition of the not insignificant portion of the population who do not drive a car. While we don’t believe this change is so great as to adversely affect the properties of the scale, the results as described herein were achieved through use of the original wording of that item, which specifically addresses driving a car. Confidence in our findings will be increased when replicated in an independent cohort.

## Conclusion

A shortened version of the NDI, the NDI-5, has been developed through qualitative and quantitative means. The scale possesses measurement properties similar to the original NDI while offering the advantage of brevity. The two symptom-based items of the original, as well as sleep, reading, and lifting have been removed for either conceptual (pain, headaches, sleep) or statistical (reading, lifting) reasons. The NDI-5 also performs similarly to the NDI-8 described by van der velde and colleagues [5] although the latter may be slightly more sensitive to change over 1 month of physiotherapy treatment. The suggestion put forth is that, for those wishing to adopt the NDI-5, concurrent use of a symptom-specific scale is encouraged to allow separation of these important constructs while maximizing sensitivity to change. Consideration should also be given to sleep-specific and/or patient-centred scales. Measurement of neck-related function appears to be a sound direction for future research considering the frequency and burden of the condition.

## Competing interests

The authors declare that they have no competing interests.

## Authors’ contributions

DW assembled the data, conducted the first-pass conceptual analysis, conducted the statistical analyses, wrote the first draft of the paper, and provided final approval. JM conducted the second-pass conceptual analysis and collaborated with DW to reach consensus, reviewed results of the statistical analysis, critically reviewed the manuscript and provided final approval. Both authors read and approved the final manuscript.

## Supplementary Material

Additional file 1 Transformation matrix. Click here for file
