# Supplementary material for: A brief 5-item version of the Neck Disability Index shows good psychometric properties
Source: Health Qual Life Outcomes. 2013 Jul 1;11:108. doi: 10.1186/1477-7525-11-108 (PMC3718697; doi:10.1186/1477-7525-11-108)
Supplement: Additional file 1 — Transformation matrix. [file 1477-7525-11-108-S1.docx]

**NECK DISABILITY INDEX - 5**

This questionnaire has been designed to give the physiotherapist information as to how your neck pain has affected your ability to manage in everyday life. Please answer every section and mark in each section only the **ONE** box which applies to you. We realize you may consider that two of the statements in any one section relate to you, but please just mark the box which most closely describes your problem over the last 24 hours.

Person care

- I can look after myself normally without causing extra pain
- I can look after myself normally but it causes extra pain
- It is painful to look after myself and I am slow and careful
- I need some help but manage most of my personal care
- I need help every day in most aspects of self care
- I do not get dressed, I was with difficulty and stay in bed

Concentration

- I can concentrate fully when I want to with no difficulty
- I can concentrate fully when I want to with slight difficulty
- I have a fair degree of difficulty in concentrating when I want to
- I have a lot of difficulty in concentrating when I want to
- I have a great deal of difficulty in concentrating when I want to
- I cannot concentrate at all

Work

- I can do as much work as I want to
- I can only do my usual work, but no more
- I can do most of my usual work, but no more
- I cannot to my usual work
- I can hardly to any work at all
- I can’t do any work at all

Driving / Riding in a vehicle

- I can drive/ride in a vehicle as long as I want without any neck pain
- I can drive/ride in a vehicle as long as I want with slight pain in my neck
- I can drive/ride in a vehicle as long as I want with moderate pain in my neck
- I can’t drive/ride in a vehicle as long as I was because of pain in my neck
- I can’t drive/ride in a vehicle at all

Recreation

- I am able to engage in all of my recreation activities with no neck pain at all
- I am able to engage in all of my recreation activities with some pain in my neck
- I am able to engage in most, but not all, of my usual recreation activities because of my neck pain
- I am able to engage in a few of my usual recreation activities because of pain in my neck
- I can hardly do any recreation activities because of pain in my neck
- I can’t do any recreation activities at all

**Appendix A: Transformation matrix**

| Raw Ordinal score /24 | Linear score /24 | Linear score /50 |
| --- | --- | --- |
| 0 | 0 | 0 |
| 1 | 4 | 8 |
| 2 | 6 | 13 |
| 3 | 8 | 16 |
| 4 | 9 | 19 |
| 5 | 10 | 20 |
| 6 | 11 | 22 |
| 7 | 11 | 23 |
| 8 | 12 | 25 |
| 9 | 12 | 26 |
| 10 | 13 | 27 |
| 11 | 14 | 28 |
| 12 | 14 | 29 |
| 13 | 15 | 30 |
| 14 | 15 | 31 |
| 15 | 16 | 33 |
| 16 | 16 | 34 |
| 17 | 17 | 35 |
| 18 | 17 | 36 |
| 19 | 18 | 37 |
| 20 | 19 | 39 |
| 21 | 19 | 40 |
| 22 | 20 | 43 |
| 23 | 22 | 46 |
| 24 | 24 | 50 |
